# A Reconfigurable Microfluidics Platform for Microparticle Separation and Fluid Mixing

**DOI:** 10.3390/mi7080139

**Published:** 2016-08-08

**Authors:** Young Ki Hahn, Daehyup Hong, Joo H. Kang, Sungyoung Choi

**Affiliations:** 1 Samsung Electronics, 4 Seocho-daero 74-gil, Seocho-gu, Seoul 06620, Korea; hahnv79@gmail.com; 2 Department of Biomedical Engineering, Kyung Hee University, Yongin-si, Gyeonggi-do 17104, Korea; hdh9080@gmail.com; 3 Department of Biomedical Engineering, School of Life Science, Ulsan National Institute of Science and Technology (UNIST), 50 UNIST-gil, Ulsan 44919, Korea

**Keywords:** reconfigurable microfluidics, inertial microfluidics, microparticle separation, fluid mixing

## Abstract

Microfluidics is an engineering tool used to control and manipulate fluid flows, with practical applications for lab-on-a-chip, point-of-care testing, and biological/medical research. However, microfluidic platforms typically lack the ability to create a fluidic duct, having an arbitrary flow path, and to change the path as needed without additional design and fabrication processes. To address this challenge, we present a simple yet effective approach for facile, on-demand reconfiguration of microfluidic channels using flexible polymer tubing. The tubing provides both a well-defined, cross-sectional geometry to allow reliable fluidic operation and excellent flexibility to achieve a high degree of freedom for reconfiguration of flow pathways. We demonstrate that microparticle separation and fluid mixing can be successfully implemented by reconfiguring the shape of the tubing. The tubing is coiled around a 3D-printed barrel to make a spiral microchannel with a constant curvature for inertial separation of microparticles. Multiple knots are also made in the tubing to create a highly tortuous flow path, which induces transverse secondary flows, Dean flows, and, thus, enhances the mixing of fluids. The reconfigurable microfluidics approach, with advantages including low-cost, simplicity, and ease of use, can serve as a promising complement to conventional microfabrication methods, which require complex fabrication processes with expensive equipment and lack a degree of freedom for reconfiguration.

## 1. Introduction

Microfluidics is a technology capable of controlling and transferring small quantities of liquids, ranging from nanoliters to microliters, which enables multiple biological assays and high-throughput screening. Particle separation and fluid mixing are important in biochemical and clinical applications for the identification and analysis of specific target molecules or cells. To achieve this, microfluidic channels have been integrated into miniaturized (lab-on-a-chip) and point-of-care testing devices as indispensable components with various advantages, such as ensuring precise control of a fluid and reducing the time and costs associated with routine biological analysis [1]. In particular, microfluidic technologies play a key role in the separation of cells or microparticles and the rapid mixing of fluids, which have been demonstrated with microfabricated, complicated structures, such as a chaotic mixer, serpentine channels, herringbone structures, and a contraction-expansion array channel [2,3,4,5,6,7,8].

Inertial microfluidics is a useful technique that exploits a unique physical phenomenon in microchannels, which utilizes the inertia of fluid flows at intermediate ranges of the Reynolds number (*Re*) (1 < *Re* < 100). This technique enables high throughput separation of microparticles and the mixing of fluids without mechanical or electrical assistance. Inertial microfluidics-based separation and mixing have shown unique utility in various applications, such as inertial focusing, ordering, and separation of microparticles and blood cells [9], DNA [10], bacterial cells [11,12], and tumor cells [13]. Most microfluidic devices, including devices for inertial microfluidics, are fabricated by conventional photolithography and soft lithography processes based on polydimethylsiloxane (PDMS) [14]. Although the fabrication process is now well established and widely used, there are several limitations to the process in terms of design flexibility and user-friendliness. First, changing the device application inevitably accompanies the modification of microfluidic devices with new designs and dimensions. For example, microfluidic mixers typically require sudden changes in flow paths to achieve efficient mixing [15], and repetitive microfluidic patterns often have to be formed for sorting microparticles [16]. Microfluidic methods, thus, require adjustment of channel geometries, according to changing target applications, for different sizes of microparticles and cells [17]. Although microfluidic devices fabricated using 3D printing technologies have been recently introduced, with the advantages of rapid prototyping and easy fabrication, these devices have a major limitation in that channel dimensions are restricted by the minimum resolution of a 3D printer [18]. The minimum resolution of 3D printers is typically in the range of tens to hundreds of micrometers, which does not permit microfabrication of fluidic channels smaller than 100 µm [19]. Recently, considerable efforts have been also made to improve the design flexibility of microfluidic platforms using modular microfluidic components [19] and punch-card-based microfluidics [20]. However, materials fabricating the channels used above are rigid, which do not allow us to adapt or change their configurations as needed. 

**Figure 1:**
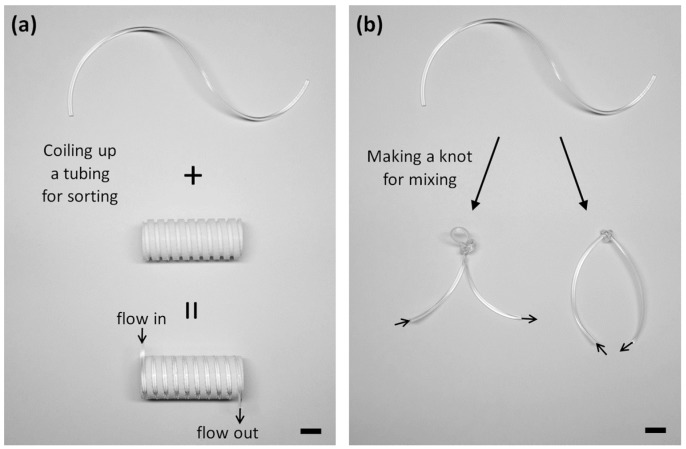
Photographs of reconfiguring flexible tubing for microparticle separation and fluid mixing. (**a**) A spiral microchannel is configured for microparticle separation by coiling the tubing onto a 3D-printed grooved barrel. (**b**) The tubing knots generate abrupt changes in a flow direction to mix laminar flows through the tubing. It shows two different ways of knotting tubing (knot with or without an inner loop) and presents that even one without microfluidic experience can construct various channel geometries by simply making knots of tubing. (scale bar: 1 cm)

To overcome these limitations, we propose a new approach to reconfigurable microfluidics to achieve facile, on-demand reconfiguration of microfluidic channels using flexible polymer tubing (Figure 1). Because flexible tubing is commonly used in most microfluidics laboratories, it is easy to access tubing components and to obtain a specific geometry of microchannels by configuring or knotting tubing without microfabrication processes. To evaluate the performance of the reconfigurable microfluidics platform, we demonstrated continuous separation of microparticles and the rapid mixing of fluids. First, we separated 15-μm and 25-μm particles with the flexible tubing coiled on a 3D-printed barrel by utilizing differential inertial focusing of particles of different sizes. Second, we confirmed that the mixing efficiency of fluids significantly improves when flowing fluids through a series of knots of flexible tubing. Thus, we successfully carried out particle separation and fluid mixing in the reconfigurable microfluidics platform, which is intended to complement existing fabrication methods for microfluidic channels and also improve the flexibility of microchannels for reconfiguration.

## 2. Materials and Methods 

### 2.1. Materials

Tygon^®^ tubing with an inner diameter (I.D.) of 190 µm and an outer diameter of 2 mm was purchased from Cole-Parmer Inc. (Vernon Hills, IL, USA). For microparticle separation, 15-µm, 20-µm, and 25-µm particles were obtained from Polysciences Inc. (Warrington, PA, USA). For fluid mixing, 100-nm fluorescent particles, purchased from Life Technologies Corp. (Carlsbad, CA, USA), were used. The particles were re-suspended in 0.1% bovine serum albumin (BSA) solution (Sigma-Aldrich Co., St. Louis, MO, USA) at a concentration of 10^4^ to 3 × 10^4^ beads/mL. BSA was used to passivate PDMS surfaces from non-specific adsorption. PDMS for microchannel fabrication was purchased from Dow Corning Inc. (Midland, MI, USA). 

### 2.2. Microfluidic Setup and Analysis 

**Figure 2:**
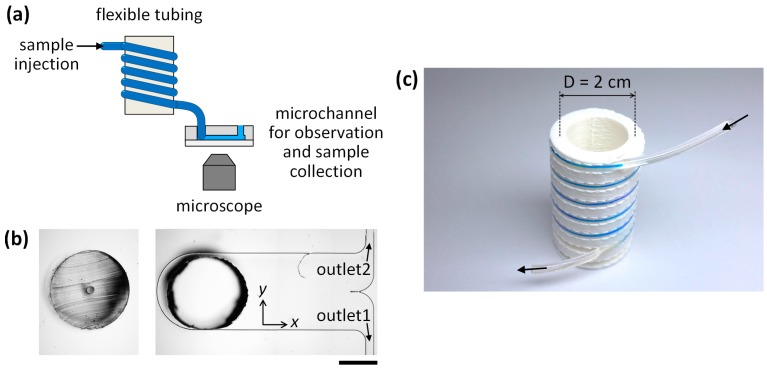
A reconfigurable microfluidics platform for microparticle separation. (**a**) Schematic showing the experimental setup for observation of the spatial distributions of microparticles after passing through the tubing. (**b**) Photographs of (**left**) a cross-section of a Tygon^®^ tubing with an inner diameter of 190 µm and an outer diameter of 2 mm, and (**right**) a microchannel for measurement of the lateral distributions of the microparticles and for the collection of separated microparticles. (**c**) Photograph of the assembled tubing (35 cm in length) onto the grooved barrel that defines a fixed radius (1 cm) of the coiled tubing. (scale bar: 1 mm)

A syringe pump (KD Scientific, Holliston, MA, USA) was used to flow sample solutions at a constant flow rate. Microfluidic behaviors were observed using a charge coupled device (CCD) camera (Nikon, Tokyo, Japan) and a high-speed camera (CASIO, Tokyo, Japan) equipped with a fluorescence microscope (Nikon) (Figure 2a). For analysis of spatial distributions of microparticles, the lateral positions of the microparticles were measured using the image analysis software, ImageJ (National Institutes of Health, Bethesda, MD, USA). After microparticle separation, the samples collected from outlet 1 and outlet 2 were analyzed using a flow cytometer (BD Biosciences, San Jose, CA, USA) (Figure 2b).

### 2.3. Fabrication of Microchannels for the Validation of a Reconfigurable Platform

A master mold for microchannels to provide fluidic access for the tubing was fabricated by standard photolithography processes, including spin-coating of photoresist, ultraviolet (UV) exposure through a photomask, development for the removal of the unexposed area of photoresist, and baking for the removal of residual solvents and solidification of the photoresist structures. Then, microfluidic channels were made by PDMS molding processes, including the mixing of PDMS with a curing agent, hardening the mixture for 1 h at 75 °C, punching to form inlet and outlet holes, and irreversible bonding between each PDMS microchannel and a glass slide. The PDMS microchannels presented in this work are intended for use in characterization of particle separation and fluid mixing and are unnecessary when used for practical applications using the reconfigurable microfluidics because other commercial tubing accessories, such as Y-shape connectors, can be simply substituted for the PDMS devices. 

### 2.4. Fabrication of a Grooved Barrel 

A grooved barrel was printed using a Mojo 3D printer (Stratasys, Eden Prairie, MN, USA). The barrel has a diameter of 2.2 cm and a spiral groove with a width of 2 mm and a depth of 2 mm, and a pitch of 6 mm (Figure 2c). The barrel is used for geometric guidance, which ensures that the tubing has a fixed radius of curvature (~1 cm) and coiling pitch. 

## 3. Results and Discussion

### 3.1. Microparticle Separation Using Coiled Flexible Tubing

**Figure 3:**
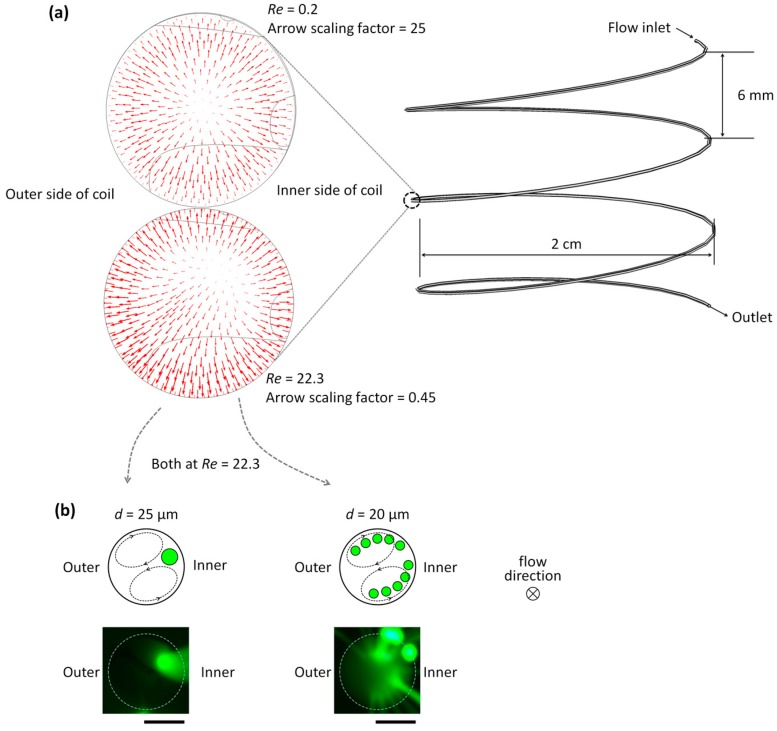
Distributions of secondary flows and the focused microparticles in a cross-section of the coiled tubing. (**a**) A computational simulation result predicts lateral flows induced by Dean flow in the coiled tubing, which results in bifurcating circulation of microparticles smaller than a critical diameter or focusing of microparticles larger than a critical diameter. Each arrow indicates a lateral flow velocity with a scale factor of 0.45 (at high *Re* of ~22) and 25 (at low *Re* of 0.2), respectively, supporting that the lateral flow is enhanced as *Re* increases. (**b**) A hypothetical diagram of the equilibrium positions of 25-µm and 20-µm particles in a cross-section of the tubing (**top**) and the corresponding experimental results (**bottom**). The photographs were taken at the end of the tubing plugged in the inlet of the microchannel for observation of microparticle distribution. Inner and outer denote the inner and outer sides of the spiral channel, respectively. The applied flow rate was 200 µL/min. (scale bars: 100 µm)

A reconfigurable microfluidics platform was applied to microparticle separation using the flexible tubing coiled onto the 3D-printed barrel, as shown in Figure 2c. Prior to the separation of microparticles, we performed a computational simulation (COMSOL Multiphysics^®^, 5.1, COMSOL Inc., Burlington, MA, USA) to predict a secondary flow induced by Dean flow in the coiled tubing, wherein rotating flows in a cross-section of the tubing were induced. The Dean flow was enhanced as *Re* increased (Figure 3a). *Re* is defined as ρ*UL*/µ, where ρ is the density of the fluid, µ is its viscosity, *U* is its average velocity, and *L* is a characteristic dimension of a channel cross-section. From the simulation results, we predicted that microparticles flowing through the coiled tubing would laterally circulate, following the Dean flow, and would be confined at a certain position where the two secondary flows bifurcate next to the inner wall surface (Figure 3b, upper left). The stability of maintaining the equilibrium position can be determined by balancing between inertial lift and Dean drag forces. Microparticles smaller than a critical size will keep the flow path following the lateral secondary flows (Dean flow), as opposed to being gathered in the equilibrium position of the coiled tubing (Figure 3b, upper right). To corroborate our prediction, 20-µm and 25-µm fluorescent particles were, respectively, injected into the coiled tubing, and their lateral position in the tubing was observed, as shown in Figure 3b. The obtained images confirmed that 25-µm particles were aligned at the equilibrium lateral position, whereas 20-µm particles were dispersed throughout the tubing. The alignment of the larger microparticles (25 µm in diameter) is attributed to the interplay between inertial lift forces and Dean drag forces. In the circular cross-section of the tubing, microparticles can migrate to the periphery of the tubing by shear-induced and wall-induced lift forces at high *Re*, which is known as the "tubular pinch" effect [21]. The coiled tubing configuration generates centrifugal forces directed outward that induce counter-rotating vortices (Dean flow). As a result of the interplay between the inertial lift and Dean drag forces, the equilibrium position of the larger microparticles can be reduced in the position right next to the inner wall of the tubing. While straight microchannels typically form multiple equilibrium positions for particles influenced by inertial lift forces at high *Re*, additional inertial forces, such as centrifugal forces and Dean drag forces, in a curved microchannel are superposed with the lift forces and can reduce particle focusing into a single stream. Because the magnitudes of the inertial lift forces and Dean drag forces highly depend on the diameter of microparticles, microparticles of different diameters can occupy different lateral positions. Due to the smaller diameter of the microparticles (20 µm in diameter), the microparticles tend to position either above or below the center line, which results in the continuous circulation of the microparticles, following the secondary lateral flows in the coiled tubing at high *Re* over 22. We note that the polystyrene microparticles (ρ = 1.04 g/cm^3^) used in this experiment have a density that is slightly higher than the surrounding medium. Their sedimentation velocity due to the gravitational force is less than 1 µm/s while their residence time in the tubing is typically less than a second. Thus, the behavior of particles is determined, not by the gravitational force, but by inertial forces. 

**Figure 4:**
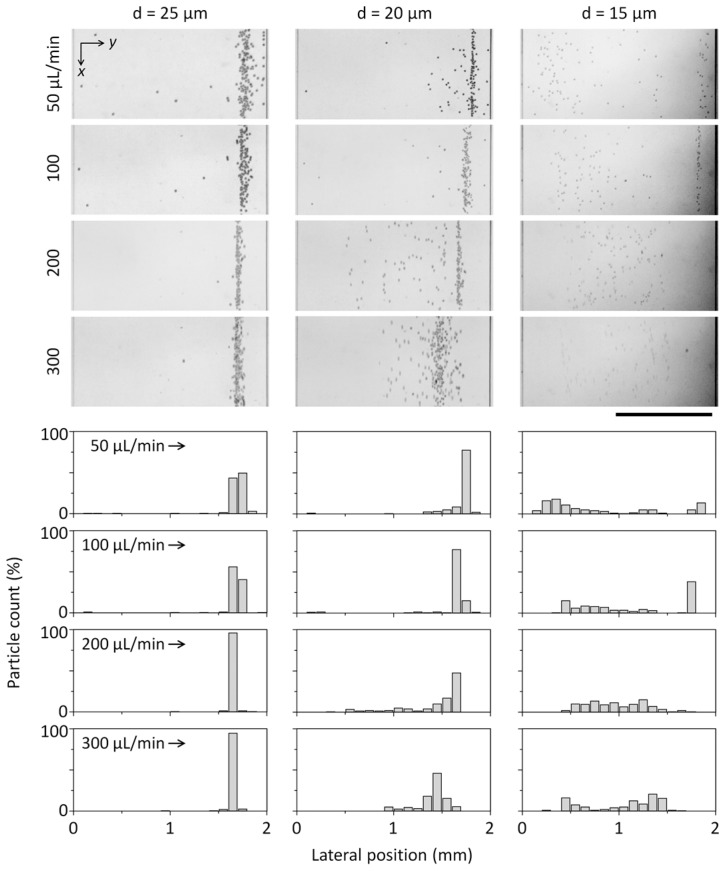
Stacked images and distribution plots show the distributions of microparticles according to the particle diameter and the flow rate. The flow direction is along the *x*-axis. (scale bar: 1 mm)

To quantitatively validate the separation capability, we connected the tubing with a microfluidic channel (2 mm in width) and measured the lateral positions of microparticles in the microfluidic channel at different flow rates ranging from 50 to 300 µL/min, which correspond to *Re* values of 5.5−33. Inertial migration of particles was first demonstrated by Segré and Silberberg in macroscale circular tubing [22]. In recent years, inertial fluidic behaviors in intermediate flow rates (1 < *Re* < 100) and microscale channels (from tens of microns to hundreds of microns in diameter) have been extensively explored in which deterministic and controllable motions of particles and fluids were observed. We also observed similar behaviors of particle migration at the intermediate *Re* regime. The equilibrium position was aligned to the right sidewall of the microchannel. The microparticles of three different diameters (15, 20, and 25 µm) were respectively flowed through the coiled tubing. As predicted, 25-µm particles coming out from the outlet of the microfluidic channels were aligned near the inner sidewall of the tubing and exited along the right sidewall of the microchannel, and the alignment of 25-µm particles was improved due to enhanced inertial lift forces as the flow rate increased to 300 µL/min (Figure 4, left-row panels). Because microparticles smaller than the critical size are more affected by the secondary flow, the particles were not confined at a certain lateral position of the tubing (Figure 4, right-row panels). The ratio of the particle diameter (*a*_p_) to the channel diameter (*D*) is a critical factor for inertial focusing in a straight pipe and needs to be *a*_p_/*D* ≥ 0.07 [23]. For *D* = 190 µm, *a*_p_ needs to be larger than 13.3 µm to satisfy the criterion. In a curved microchannel, the secondary flow, Dean flow can perturb flowing particles and affect their equilibrium position. The ratio of inertial lift and Dean drag (*R*_f_ = 2*a*_p_^2^*R*/*D*^3^) considerably increase with *a*_p_ [24] and so relatively strong Dean forces for 15-µm particles likely result in disruption of particle focusing, where *R* is a radius of curvature of the coiled tubing. In a curved microchannel, inertial particle focusing depends on the balance between inertial lift and Dean drag forces. The focusing behavior of 20 µm-particles is significantly affected by a flow rate (Figure 4) and this can be explained by increased Dean drag forces which can destabilize a preferred focusing position at high *Re* over 22. 

**Figure 5:**
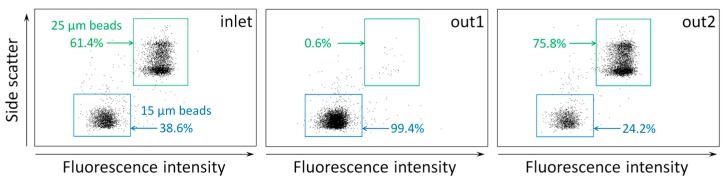
Flow cytometry results showing the separation of 15-µm and 25-µm particles. The purity of separated microparticles was analyzed using a flow cytometer. Purity is defined as the percentage of the number of target microparticles in each sorted population.

In addition to measuring the lateral positions of microparticles, we demonstrated the separation and collection of microparticles using the microchannel with two outlets connected to the coiled tubing (Figure 2b). The populations of microparticles collected from outlet 1 and outlet 2, respectively, were analyzed by a flow cytometry. The flow cytometry results support that two different sizes of microparticles (15 and 25 µm in diameter) mixed in a solution can be separated and significantly enriched when flowing through the coiled tubing with a throughput of 4.6 × 10^3^ microparticles/min. A solution containing the two different sizes of microparticles (15 µm and 25 µm with an initial ratio of 38.6% and 61.4%, respectively) was injected into the coiled tubing at a flow rate of 200 µL/min. The purity of 15-µm particles in the sample collected from outlet 1 was significantly improved to 98.5 ± 2.7%, whereas the purity of 25-µm particles in the sample from outlet 2 was 75.6 ± 4.4% (*n* = 3). This is most likely due to the continuous circulation of smaller microparticles (15 µm) by the balance between inertial lift and Dean drag forces. Additionally, the recoveries of 15-µm and 25-µm microparticles were determined to be 50.9 ± 5.3% in outlet 1 and 99.5 ± 0.9% in outlet 2 (Figure 5) (*n* = 3). Recovery is defined as the number of sorted target particles divided by the total number of the particles injected. 

### 3.2. Mixing of Laminar Flows Using Knots of Tubing

**Figure 6:**
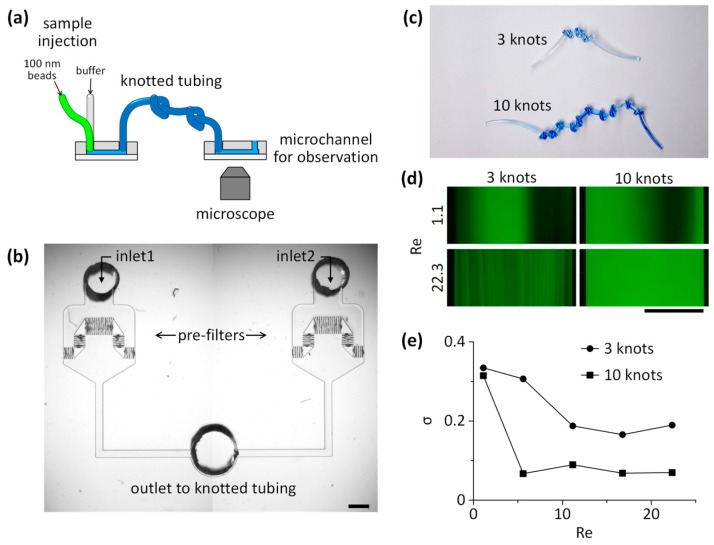
Mixing of fluids using knots of flexible tubing. (**a**) Schematic showing the experimental setup for observation of the spatial distribution of fluorescent nanoparticles after mixing. (**b**) Photograph of a microchannel for the infusion of two types of fluids to assess the mixing efficiency (one with 100 nm fluorescent nanoparticles and the other one without the particles). The outlet is directly connected to the flexible tubing with serial knots without premixing. (**c**) Photograph of knots in the tubing, which induce abrupt changes in a flow direction. (**d**) Fluorescence images after the mixing of the buffer solution (no fluorescent nanoparticles) and the solution containing 100-nm green-fluorescent nanoparticles. (**e**) The degree of mixing efficiency can be obtained from the standard deviation of the fluorescent intensity across the channel. The 100-nm fluorescent particles in laminar flows were completely mixed when flowing through 10 serial knots of tubing, even at low Reynolds numbers, in comparison to the tubing with 3 knots. (scale bars: 1 mm)

**Figure 7:**
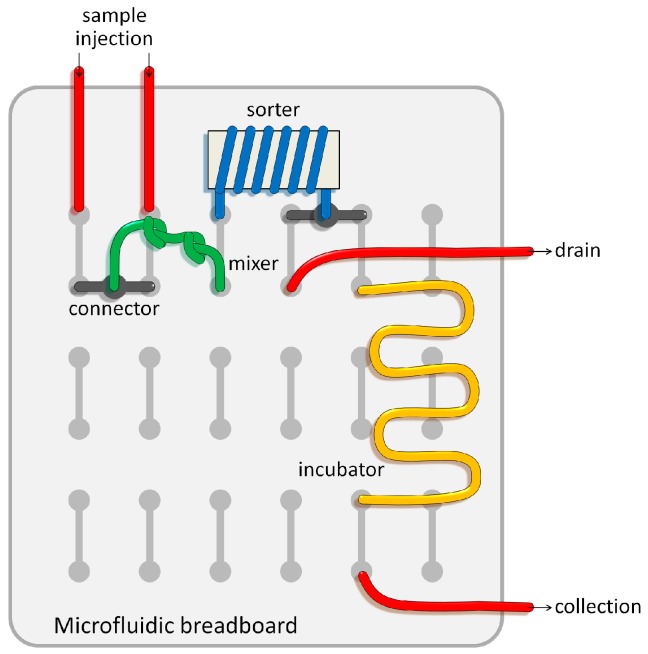
Schematic showing the potential utility of the reconfigurable microfluidics approach for a microfluidic breadboard that enables rapid testing of a prototype microfluidic circuit with modular microfluidic components.

We then demonstrated that a reconfigurable microfluidics platform could be applied to achieve high mixing efficiency with a simple configuration of tubing with a series of knots (Figure 6). Mixing efficiency was defined as a standard deviation, σ, of the fluorescence distribution. A value of 0 corresponds to complete mixing and 0.5 to complete segregation. For the mixing of fluids, multiple knots (trials with 3 and 10 knots) were made within a tubing (Figure 6c). We did not observe any structural changes in the knots during experiments. A solution containing fluorescent nanoparticles (100 nm in diameter) and the buffer solution were injected through a Y-shaped microchannel with two inlets, and the intersection where the two fluids met was directly connected to the tubing inlet without premixing in the microchannel (Figure 6b). Because the diffusion coefficient (*D*_diff_) of nanoparticles (100 nm, ~10^−8^ cm^2^/s) is about three order magnitudes lower than small fluorescent molecules (FITC, *D*_diff_ = 0.64 × 10^−5^ cm^2^/s), the Peclet number (*Pe*) of the mass transport across the two laminar flows driven by diffusion is not effective. Thus, the mixing of the nanoparticles in laminar flows requires a certain mixing component to achieve complete homogenization in fluids. Efficient mixing can be achieved by knots of tubing because the direction of the secondary lateral flow (Dean flow) is irregular and dramatically changes at every turn of knotted tubing, which causes non-uniform lateral circulatory flows, and subsequently causes effective mixing in the knotted tubing. In addition, mixing efficiency is predicted to be improved when a flow rate increases because the Dean number is proportional to the Reynolds number [25]. At low *Re* (1.1) herein, efficient mixing was not achieved, even after passing through 10 serial knots of tubing. However, when we increased *Re* to 22.3, the complete mixing of 100-nm fluorescent particles was achieved after flowing through 10 serial knots of tubing (Figure 6d). The standard deviation (σ) of the fluorescence intensity across the microchannel is plotted in Figure 6e, revealing that the number of knots of tubing is more critical to achieve complete mixing of fluids than influences of *Re*. This is because the mixing efficiency affected by *Re* plateaued above a certain flow rate. The cross-sectional deformation of the knotted tubing may occur and affect the mixing performance; however, the tubing was adequately (not too much tightly) knotted to minimize such deformation. Thus, the mixing of fluids is mainly affected by abrupt changes in a flow direction rather than subtle changes in a channel cross-section. These results support that the proposed reconfigurable microfluidics platform can simply replace conventional microfluidic components and facilitate facile integration of the components as shown in Figure 7.

## 4. Conclusions and Perspectives

We demonstrated that size-based separation of microparticles and mixing of laminar flows at low *Re* can be achieved by coiled and knotted flexible tubing. We constructed coiled tubing using a 3D-printed barrel that fixes tubing to a coiled configuration. Microparticles were laterally circulated, following the secondary flow (Dean flow) generated by the curvature of the coiled tubing, and the lateral positions of the microparticles could be confined at a certain position when the particle size was greater than 20 µm. The enrichment of microparticles was successfully demonstrated using a mixture of microparticles with diameters of 15 µm and 25 µm. The purity of 15-µm particles in outlet 1 was 98.5 ± 2.7% while the recovery rate of 25-µm particles from outlet 2 was 99.5 ± 0.9%. Because the focusing and separation of microparticles in the coiled tubing is attributed to the balance between the secondary lateral flow (Dean flow) and inertial lift forces, the critical size for separation can be determined by the relative difference between the particle diameter, the inner diameter of the coiled tubing, and the Dean number of the flow. To evaluate the versatility of the reconfiguration approach, we uncoiled the tubing and tied it to make serial knots to induce abrupt changes in a lateral flow direction and improve the mixing of fluids. The knots of tubing were made to form repeating tortuous flow paths that yield efficient mixing of laminar flows even at low *Re* (5.6–22.3). These knots of tubing induced the secondary circulating flows in irregular directions, resulting in local agitation and effective mixing of laminar flows in the tubing.

The strong point of the proposed reconfigurable microfluidics platform using flexible tubing is that it allows one even without microfabrication experiences to construct microfluidic channels for particle separation and fluid mixing, and that the shape of flexible tubing can be easily adapted to a certain configuration when required. In this study, we demonstrated the utility of the reconfigurable flexible tubing-based microfluidics platform as a substitution for the existing microfluidic components for microparticle separation and fluid mixing. More importantly, our proposed approach can be applied to the research field of microfluidic chemical synthesis, which requires proficiency in mixing and separating product particles, as well as robust reliability in enduring harsh chemical conditions (nearly impossible to achieve with conventional PDMS-based microfluidic devices due to the inherent characteristics of PDMS) [26]. The construction of a reconfigurable microfluidics system using chemical-resistant flexible tubing, such as Teflon tubing, would make the design and fabrication of a microfluidic system much more straightforward, in which a series of chemical mixing, synthesis, and size-based separation can simultaneously take place, as shown in Figure 7. The proposed platform will easily extend its applicability to chemical screening and synthesis if a microfluidic breadboard is printed in thermoplastic materials with chemical resistance using a 3D printer [19].
